# The risk of developing dementia in the COVID‐19 pandemic; a cohort study

**DOI:** 10.1002/gps.6041

**Published:** 2024-01-13

**Authors:** Daniel Hendrik Baron, Elizabeth Coulthard, Carslake David, Lindsey Isla Sinclair

**Affiliations:** ^1^ Dementia Research Group University of Bristol Bristol UK; ^2^ Frimley Health NHS Foundation Trust Surrey UK; ^3^ ReMemBr Group University of Bristol Bristol UK; ^4^ Population Health Sciences University of Bristol Bristol UK; ^5^ MRC Integrative Epidemiology Unit University of Bristol Bristol UK

**Keywords:** Alzheimer's disease, cognitive dysfunction, COVID‐19, mild cognitive impairment, quarantine, sars‐CoV‐2, social isolation

## Abstract

**Objectives:**

The effects of the COVID‐19 pandemic on cognitive decline are not fully understood. Higher social activity and relationships have been associated with decreased risk of dementia. We hypothesised that risk of transition to dementia would increase after the start of the first national lockdown.

**Methods:**

We obtained data from the Brains for Dementia (BDR) cohort, which has collected roughly annual data on 3726 older adults with and without dementia since 2008. Data continued to be collected during the lockdowns, although by telephone and/or video call instead of in person. Individuals diagnosed with dementia at study entry were excluded from this study as were individuals with only one visit. Cognitive status was classified using the Clinical Dementia Rating (CDR) global score. Poisson regression with cubic splines to account for differences in age was used to compare the incidence of dementia before and after March 1st 2020.

**Results:**

Out of 2242 individuals, 208 individuals developed dementia before and 50 developed dementia after 01/03/20. The incidence rate ratio of developing dementia after 01/03/20 was 0.847 (0.538–1.335) *p* = 0.570. In our secondary analysis we found that the positive association between mild cognitive impairment (MCI) and dementia incidence decreased after 1/3/20 (interaction effect *p* = 0.031).

**Conclusion:**

The incidence of dementia as defined using the CDR global score did not change significantly after the first lockdown began, but we found evidence that lockdown decreased the positive association between MCI and dementia incidence. This may reflect that individuals were progressing to dementia more rapidly and thus missing the MCI stage or that assessing patients over the phone made diagnosing MCI more challenging.

## Key points

- Large cohort study with data collected both before and during the COVID‐19 pandemic.
- The incidence of dementia did not appear to have increased during the pandemic.
- The protective measures during the COVID‐19 pandemic may have led to a more rapid decline in cognition in people who developed dementia.

## 1 INTRODUCTION

Dementia is a major cause of death and disability worldwide. There is increasing evidence that some cases of dementia may be preventable, for example, by improved control of blood pressure in mid‐life.^1^ The global prevalence of dementia in all age groups is predicted to increase by 117% between 2016 and 2050, which is a similar percentage increase to between 1990 and 2016.^1^ From 2016, mainly middle‐ and low‐income countries will be affected and will see a huge shift in the age structure of the population.^1^

Mild cognitive impairment (MCI) is thought to be the intermediate stage between having a normal cognitive function and dementia.^2^, ^3^ Increasingly, research is focussing on aiming preventative measures at the earliest stages of dementia.^3^ In 2020, the findings of an update to the *Lancet* Commission on dementia prevention, intervention and care suggested that 12 modifiable risk factors account for around 40% of the risk of developing dementia. One of these 12 modifiable risk factors for dementia is social isolation with an estimated relative risk for dementia of 1.6 (95% confidence interval (CI) 1.3–1.9) in people aged over 65 years old.^4^ Other risk factors identified included depression, hearing loss, diabetes, hypertension, physical inactivity, obesity, alcohol consumption and air pollution. The potential for prevention by targeting these 12 risk factors is high particularly in middle‐ and low‐income countries.^4^ As the finances available in these countries are limited, prioritisation is key.^5^

The annual conversion rate (ACR) of MCI to dementia in studies with a short‐term follow‐up period (<2 years) is reported as 10%–15%, with a recent study reporting 18.4% conversion over one year.^6^ In studies using long‐term follow‐up (5–10 years), however, the ACR is reduced to approximately 3%–5%.^7^, ^8^ The difference in short‐term and long‐term ACR may be explained by the heterogenous group of MCI patient selection when conducting a study, consisting of a varying disease entity. The labelling of, for example, early dementia as MCI will influence how quickly that person will progress to dementia.^8^

There are no currently licenced treatments to prevent dementia progression or progression from MCI to dementia. People with MCI are recommended to reduce alcohol consumption, to do regular exercise (at least twice per week) including aerobic exercise, to undergo cognitive interventions,^9^, ^10^ such as group‐based exercises with or without computers^11^ and to have social interactions on a daily basis.^12^ The COVID‐19 pandemic introduced multiple factors that could have hastened progression of dementia, including reduced social interaction, increases in problem drinking, and reductions in physical activity and healthy eating in some but not all studies.^13^, ^14^, ^15^, ^16^ Throughout the pandemic, the level and severity of the protective measures varied, even during the three periods of national lockdowns in the UK^17^ and there is only low‐quality evidence of the effect of social interventions on dementia.^4^ In general people with dementia were more likely to take a careful approach such as isolating compared to people without dementia during the pandemic.^18^ Interestingly, the DETERMIND group reported evidence that people with dementia experienced increased or decreased level of loneliness during the ‘first’ lockdown based on the status of their carer.^19^

The ‘social distancing’ strategies, including voluntary isolation and 2‐m distance, used during the pandemic to limit the spread of the virus by interrupting viral transmission through physical contact^20^ may have worsened cognitive decline but at the same time may have helped to prevent it by, for example, reducing COVID‐19 infections. A study at the beginning of the pandemic suggested that COVID‐19 infection may worsen neuropsychiatric symptoms in older adults with or without dementia.^21^ These suggestions are supported by current literature reporting that COVID‐19 infections can have long‐term detrimental effects on cognitive function.^22^, ^23^

Therefore, more information is required on the effects of the COVID‐19 pandemic on the development of dementia and progression of cognitive decline. New evidence will allow for a better understanding of the impact of the COVID‐19 pandemic on cognitive decline and ultimately can be used to aid prevention. Our study aims to look at the effects of the COVID‐19 pandemic on the transition from normal cognition to dementia using a large cohort. We hypothesised that all the changes within British society such as the protective measures in response to the COVID‐19 pandemic would lead to an increased transition to dementia compared to before the start of the pandemic.

## 2 METHODS

Data were obtained from Brains for Dementia Research (BDR), a cohort which has collected approximately annual data from 3112 older adults with and without dementia since 2008. For details on the BDR cohort including entry and exclusion criteria see supplementary Table S1.^24^ Visits are conducted by a trained nurse or psychologist^25^ using standardised data collection proformas. Even before lockdown some appointments were conducted by telephone (e.g. control appointments) and data collection by telephone and/or video call continued through lockdown. Data collection in person was resumed after the protective measures were lifted in mid 2021. BDR appointments for controls have always been by telephone, visits to those with cognitive symptoms were in person prior to the lockdown and after mid 2021 and visits to those with severe dementia were carried out by interviewing carers. Whilst the BDR cohort is better educated than the general population, ethnicity and sex are representative of the UK older adult population.

Cognitive status was classified at visits by the assessing nurse or psychologist using the Clinical Dementia Rating (CDR) global score, developed to measure the severity of dementia (supplementary Table S2).^24^ For the purposes of our analysis, we defined normal cognition as 0, MCI as 0.5 and dementia as 1–3. The CDR sum of boxes is a related score which tests both cognitive and functional aspects of dementia^26^ and scores range between 0 and 18. In a sensitivity analysis, we instead defined dementia using CDR sum of boxes as normal cognition (0), MCI (0.5–2.5) or dementia (3–18). Due to missing data, it was not possible to define dementia using either the Mini Mental State Examination (MMSE) or Montreal Cognitive Assessment (MoCA) see supplementary Table S4. Visits also included the collection of data on a wide range of variables including comorbidities (diabetes, hypertension, cancer, etc.), drinking and smoking status, accommodation, and prescribed medication.

**FIGURE 1:**
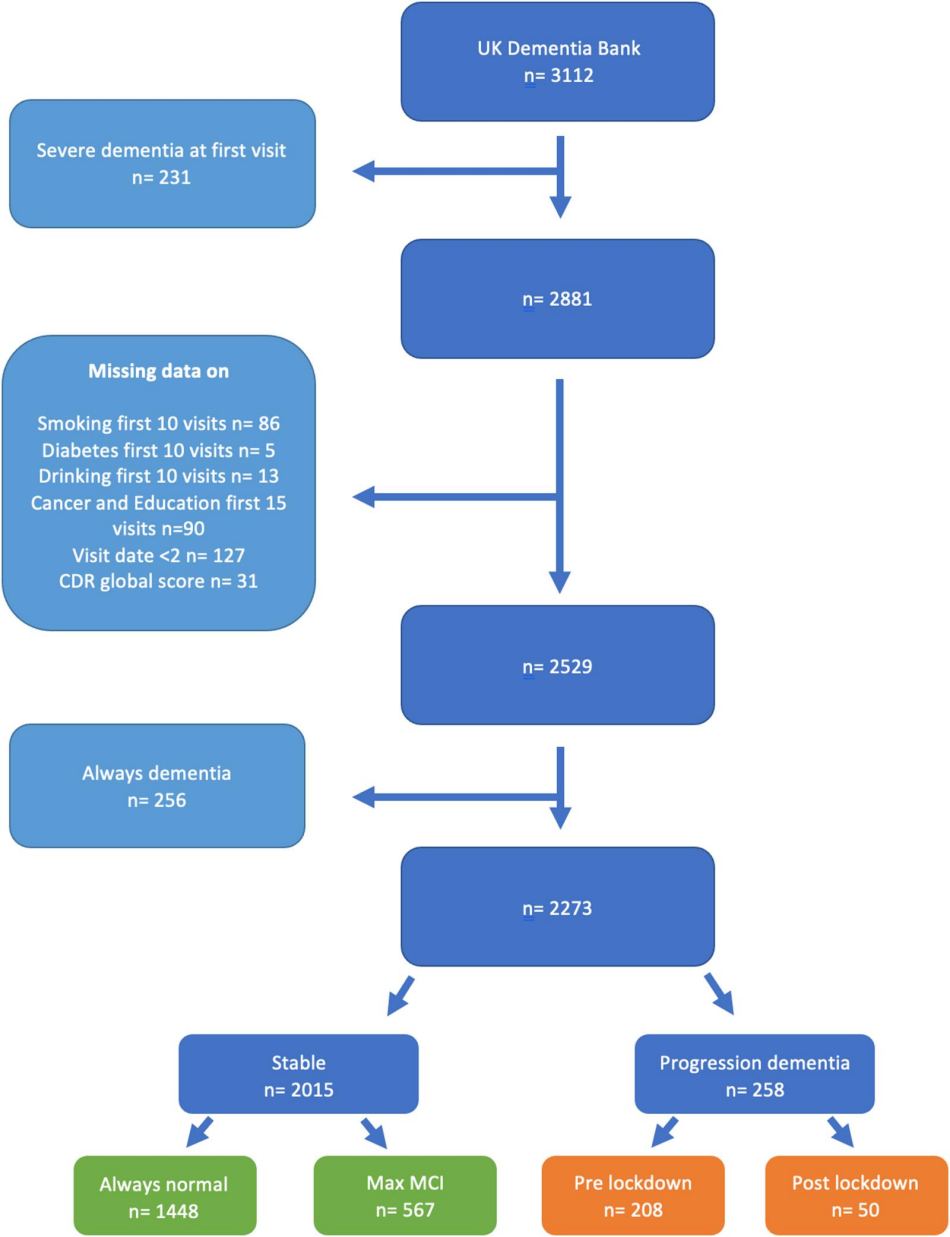
Flowchart of patient selection and exclusion criteria per group. ‘Always normal’ = all the patients that had a normal cognition throughout the data collection period, ‘Always dementia’ = all the patients with a Clinical Dementia Rating (CDR) global score ≥1 at first visit, ‘Severe dementia at first visit’ = all the patients with CDR global score = 3 at first visit.

Individuals diagnosed with dementia at or prior to study entry were excluded from this study as were individuals with only one visit and missing CDR (global) scores. For details see Figure 1. Summary variables were calculated as the maximum or worst outcome reported (e.g. heaviest smoking) in the first 10 years of follow‐up.

Descriptive statistics were used to describe the baseline characteristics of the study sample including age, smoking according to their outcome (always normal, MCI only, dementia incidence before lockdown or dementia incidence after lockdown). For details see Supporting Information S1.

The event of interest was a person's first transition from a normal or MCI cognitive state to a state of dementia. The cognitive state, including dementia, was assessed during the first visit using a range of cognitive tests and questionnaires including the CDR and the neuropsychiatric inventory. Each person's follow‐up time began at their first visit and continued until the visit when they were first found to have dementia, or (if they were never found to have dementia) their last visit before the data freeze for this study on 8th May 2022. Because the exact time of the event was unknown, follow‐up was divided into the intervals between visits. The exposure of interest was the interval's lockdown status. An interval was defined as a pre‐lockdown interval (scored 0) if it ended before 1st March 2020 and a post‐lockdown interval (scored 1) if it began after this date. The formal lockdown in the UK started on March 16th to 17th but many vulnerable people had been isolating for a significant period prior to the formal lockdown and COVID‐19 had already reached the UK. Intervals containing 1st March 2020 were given a value corresponding to the proportion of the interval that took place after this date and the exposure was treated as a continuous variable. In a sensitivity analysis, such intervals were instead defined as peri‐lockdown intervals, given a value of 2, and the exposure was treated as categorical.

The data available from the study to researchers is updated every week and we used data from the 8th May 2022 data freeze. The data was provided in a single Excel file which was imported into Stata 17 software.^27^ Following data cleaning the data was coded as a wide dataset for generation and summary variables and then converted to a long dataset for the Poisson regression analysis.

The primary outcome was the conversion rate of normal or MCI cognitive state to dementia before and after the lockdown using the CDR global score. BDR is a longitudinal cohort and was unusual in continuing to collect data throughout the pandemic. The COVID‐19 pandemic can be viewed as the ‘exposure’. As the pre‐lockdown period is longer (2008–2020) than the period post‐lockdown period (2020–2022), it is impossible to recruit a 1:1 power ratio as these two periods would need to be a similar length of time.

Secondary outcomes included dementia as defined using the CDR sum of boxes and risk analysis of converting to dementia following MCI or normal cognition (defined using CDR global) before and after the lockdown.

We used a Poisson regression on the data split into intervals, to assess the association between lockdown status and dementia incidence. The outcome was the person's dementia status at the end of the interval and the natural logarithm of the interval duration was used as an offset. In intervals ending with dementia, the duration was halved (representing an assumption that the event took place at the interval's mid‐point). Age is a very important risk factor for dementia,^28^ and inevitably associated with our exposure. To best account for it we adjusted for the person's age at the interval's mid‐point, added to the model as a cubic spline with five knots positioned at the recommended percentiles.^29^ See Supporting Information S1 for risk factor adjustments.

To investigate the effect of MCI on dementia incidence before and after lockdown, a binary term indicating whether the person had MCI at the beginning of the interval was added and allowed to interact with the exposure (Supporting Information S1 for more detail).

## 3 RESULTS

A total of 3112 individuals accounting for 16,459 visits were included in the study. After the exclusion criteria were applied, 2273 eligible individuals remained (see Figure 1). Out of these 2273 individuals, 208 individuals developed dementia before and 50 developed dementia after 01/03/2020. The median interval between visits throughout the study was 425 days with an interquartile range of 355 days. The interval between visits was always ≤2 years for 80.0% of participants.

Individuals who had a CDR of 1 (i.e., categorised as dementia) during the study were older on average at baseline, more likely to be male and to have had a stroke (see Table 1). See Table 2 for additional information regarding cognitive function at the first visit whereby CDR global score = 1.

**TABLE 1:** Baseline demographic and clinical information on the individuals included in this study.

	Always normal	Max of MCI	Progression dementia pre lockdown	Progression dementia post lockdown	Missing data	*p* value (Chi^2^/ANOVA/KW test)
Number of participants	1448	567	208	50	‐	
Age	73.2	77.2	78.8	80.3	‐	0.000
	(6.9)	(7.6)	(9.3)	(5.7)		(41.54)
Gender						0.000
						(26.37)
Female	975	358	103	30	‐	
	67.33%	63.14%	49.52%	60.00%		
Male	473	209	105	20	‐	
	32.67%	36.86%	50.48%	40.00%		
Education	14.2	13.3	13.1	12.7	‐	0.639
	(3.4)	(3.5)	(3.6)	(3.2)		(1.69)
CDR sum of boxes	0.0	0.3	1.0	0.3	‐	<0.001
	(0.1)	(0.5)	(0.9)	(0.5)		(53.47)
BADL	0.5	1.4	5.4	1.0	1372	<0.001
	(2.8)	(3.2)	(6.2)	(1.8)		(61.50)
HICS	0.6	1.9	2.6	1.6	1651	<0.001
	(0.9)	(2.2)	(2.2)	(1.4)		(53.47)
MMSE	29.3	28.7	26.8	27.9	194	<0.001
	(1.4)	(1.8)	(3.2)	(2.5)		(239.38)
MOCA	26.9	24.8	23.0	25.3	1042	<0.001
	(3.3)	(3.8)	(4.1)	(2.9)		(144.10)
TICS	29.7	25.0	25.0	.	2247	0.794
	(3.6)	(.)	(2.8)	(.)		(0.07)
Geriatric depression scale	1.4	2.2	2.9	2.1	168	<0.001
	(1.8)	(2.3)	(2.5)	(2.0)		(128.36)
Smoking					‐	0.401
						(6.21)
10–20/day (for a year or more)	293	127	7	14		
	20.23%	22.40%	23.60%	28.00%		
Heavy smoker (at least 20/day for a year or more)	165	69	16	6		
	11.40%	12.17%	7.69%	12.00%		
No or less than 10/day	990	371	145	30		
	68.37%	65.43%	69.71%	60.00%		
Drinking					‐	0.834
No	1275	492	187	42		
	88.05%	86.77%	89.90%	84.00%		
Yes	172	75	21	8		
	11.88%	13.23%	10.10%	16.00%		
Diabetes					‐	0.044
No	1288	475	181	42		
	88.95%	83.77%	87.02%	84.00%		
Yes	160	91	27	8		
	11.05%	16.05%	12.98%	16.00%		
Marital status					38	0.000
						(48.10)
Divorced/single	261	99	16	5		
	18.34%	15.92%	7.84%	10.20%		
Married	690	232	99	13		
	48.49%	41.50%	48.53%	26.53%		
Widowed	470	234	88	31		
	33.03%	41.86%	43.14%	63.27%		
Accommodation					287	0.000
						(81.62)
Living alone	507	211	53	19		
	39.24%	44.61%	29.94%	43.18%		
Living with family/friend/other	49	19	11	5		
	3.79%	4.02%	6.21%	11.37%		
Living with spouse/cohabite	724	231	98	19		
	56.04%	48.84%	55.37%	43.18%		
Family history					‐	0.004
						(13.33)
No	1095	468	169	41		
	75.62%	82.54%	81.25%	82.00%		
Yes	353	99	39	9		
	24.38%	17.46%	18.75%	18.00%		
Memory problem					1907	
No	267	61	10	10		
	100%	92.43%	50%	76.92%		
Yes	0	5	10	3		
	0%	7.57%	50%	23.08%		
Sensory impairment					619	0.000
						(59.65)
Normal hearing	842	255	74	21		
	78.11%	61.30%	60.16%	56.76%		
Normal hearing with aid	115	79	22	9		
	10.67%	18.99%	17.89%	24.32%		
Impaired	121	82	27	7		
	11.23%	19.71%	21.96%	18.92%		
Cancer					‐	0.000
No	1019	373	145	34		
	70.37%	65.78%	69.71%	68.00%		
Yes	428	194	63	15		
	29.56%	34.22%	30.29%	30.00%		
SARS‐CoV‐2 infection					‐	0.705
						(1.40)
No	1415	553	201	48		
	97.72%	97.53%	96.63%	96.00%		
Yes	33	14	7	2		
	2.28%	2.47%	3.37%	4.00%		
SARS‐CoV‐2 vaccine					‐	0.206
						(8.47)
No	1104	471	197	40		
	76.24%	83.07%	94.71%	80.00%		
Yes	344	96	11	10		
	23.76%	16.93%	5.29%	20.00%		
Head injury					‐	0.172
						(0.903)
No	1231	456	168	40		
	85.01%	80.42%	80.77%	80.00%		
Yes	216	110	40	10		
	14.92%	19.40%	19.23%	20.00%		
Stroke					6	0.000
						(52.33)
No	1366	500	169	43		
	94.60%	88.18%	82.04%	86.00%		
Yes	78	67	37	7		
	5.40%	11.82%	17.96%	14.00%		
Hypertension					‐	0.004
No	650	200	86	15		
	44.89%	35.27%	41.35%	30.00%		
Yes	797	366	122	35		
	55.04%	64.55%	58.65%	70.00%		
Heart attack					‐	0.015
No	1342	496	185	45		
	92.68%	87.48%	88.94%	90.00%		
Yes	103	70	23	5		
	7.11%	12.35%	11.06%	10.00%		

*Note*: Information on COVID‐19 infection and vaccination status was taken from 2020 to 2022 as this data was not available as baseline. Data presented are numbers (percentages) or mean (SD). “Always normal” = the group of patient with a normal cognition throughout the data collection phase.

Abbreviations: ANOVA, Analysis of Variance test; Chi2, chi‐squared test; KW, Kruskal Wallis test.

**TABLE 2:** Cognitive function reported memory problems and clinical diagnoses at the first visit where CDR global = 1.

	Dementia pre lockdown (*n* = 208)	Dementia post lockdown (*n* = 50)
Memory problems reported		
No (%)	18 (8.6)	2 (4.0)
Yes (%)	23 (11.0)	8 (16.0)
Missing data (%)	167 (80.4)	40 (80.0)
Clinical diagnosis		
AD/PCA/PPA/mixed dementia (%)	45 (21.6)	7 (14.0)
Unknown (%)	64 (30.8)	8 (16.0)
Missing data (%)	99 (47.6)	35 (70.0)
Diagnosed with dementia		
Yes (%)	157 (75.5)	20 (40.0)
No, or unknown (%)	51 (24.5)	30 (60.0)
MMSE score		
Mean (SD)	21.8 (5.2)	22.8 (3.7)
Missing data *n* = (%)	23 (11.1)	25 (50.0)
MoCA score		
Mean (SD)	18.4 (5.1)	16.0 (6.2)
Missing data *n* = (%)	162 (77.9)	43 (86.0)

*Note*: Data was not available on all measures for all individuals with dementia. Data presented are numbers (percentages) or mean (SD).

Abbreviations: AD, Alzheimer's Dementia; MMSE, Mini Mental State Examination; MoCA, Montreal Cognitive Assessment; PCA, Posterior Cortical Atrophy; PPA, Primary Progressive Aphasia.

The primary analysis, as shown in Table 3, did not show a significant effect of the COVID‐19 pandemic on the incidence of dementia. In our secondary analysis, however, when using the CDR sum of boxes there was a significant effect on the incidence of dementia, see Table 3. We also found some evidence that the positive association between MCI and dementia incidence at the next follow‐up visit was weaker following lockdown compared to before lockdown, see Table 4. The IRR was approximately halved if the age effect was held constant and reduced fourfold if it was allowed to change, although these estimates were imprecise. Neither result was greatly affected by adjustment for other covariates. The independent effect of the risk of the covariates in our analysis is shown in supplementary Table S3. MCI is widely considered to be a transitional stage between normal cognition and dementia, although some individuals do revert to normal cognition from MCI.^30^ It was not possible to include all the covariates used in the primary analysis as the Poisson model was unable to converge. We therefore removed covariates, starting with those that did not differ between our groups in order of dementia risk, until the model converged. The effect of age in the secondary and primary analyses is shown in supplementary Figures S1 and S2.

**TABLE 3:** The incidence of dementia after versus before March 1st 2020.

Method for dementia definition	Adjusted only for current age		Adjusted for age at study entry, sex, SARS‐CoV‐2 infection, stroke, hypertension and diabetes		+ current marital status & current sensory impairment	
	IRR (95% CI) post‐ versus pre‐ lockdown	*p*	IRR (95% CI) post‐ versus pre‐ lockdown	*p*	IRR (95% CI) post‐ versus pre‐ lockdown	*p*
CDR global score	0.847 (0.538–1.335)	0.474	0.862 (0.520–1.431)	0.567	0.856 (0.507–1.449)	0.563
CDR sum of boxes	1.438 (1.012–2.045)	0.043	1.606 (1.061–2.31)	0.025	1.560 (1.017–2.394)	0.042

*Note*: In the primary analysis dementia was defined using the CDR global score ≥1 and in the secondary analysis it was defined using the CDR sum of boxes ≥3.

**TABLE 4:** The incidence of dementia among individuals with MCI compared to normal cognition at the previous visit.

	Adjusted only for current age		Adjusted for current age + sensory impairment, sex, SARS‐CoV‐2 infection, and marital status	
	IRR (95% CI) MCI versus normal cognition	*p*	IRR (95% CI) MCI versus normal cognition	*p*
Model allowing effect of age to differ pre/post lockdown				
Before lockdown	50.523 (28.740–88.813)	<0.001	45.197 (23.155–88.221)	*p* < 0.001
After lockdown	13.433 (4.622–39.041)	*p* < 0.001	11.913 (3.969–35.753)	*p* < 0.001
Test for interaction between MCI and lockdown		*p* = 0.031		*p* = 0.038
		chi^2^ = 4.63		chi^2^ = 4.31
Model allowing common effect of age pre/post lockdown				
Before lockdown	35.806 (22.373–57.303)	*p* < 0.001	30.389 (17.651–51.976)	*p* < 0.001
After lockdown	17.012 (6.043–47.889)	*p* < 0.001	14.559 (5.100–41.562)	*p* < 0.001
Test for interaction between MCI and lockdown		*p* = 0.197		*p* = 0.220
		chi^2^ = 1.66		chi^2^ = 1.50

*Note*: The covariates used in this model differ from the primary analysis as with those co‐variates included the Poisson regression model was unable to converge. Peri‐lockdown periods were only included in the model allowing a common effect of age pre/post lockdown.

Defining MCI purely by CDR global score is potentially problematic, so we also examined whether individuals with MCI reported memory problems at the visits at which their CDR global score was equal to 0.5. For individuals who had a CDR global score of 0.5 at a visit 24.0% reported memory problems, the mean MMSE score was 27.2 (ranging from 7 to 30, missing for 214 participants), the mean MoCA score was 23.2 (ranging from 9 to 30, missing for 611 participants) and 35.3% had a diagnosis of a neurodegenerative illness.

## 4 DISCUSSION

Using a large UK based cohort focussing on dementia we have studied the effect of the UK COVID‐19 pandemic including the protective measures on the risk of an individual developing dementia. Only 50 individuals developed dementia after exposure to the protective measures during the COVID‐19 pandemic, which reduces the power of this study. Because this study was unusual in continuing to collect data during the COVID‐19 pandemic it was not possible to include additional cohorts to increase the study power. Despite the relatively low power of this study, we found some evidence in our secondary analysis with an alternative outcome definition that exposure to the COVID‐19 pandemic may have increased the risk of older adults developing dementia, although this was not seen in our primary analysis. We also found evidence that the effect of MCI appeared to be lower post lockdown although this was not demonstrated in all our statistical models. This may represent more rapid deterioration in cognition in which individuals deteriorated too quickly between visits to be assessed as having MCI at annual follow‐up instead of dementia. The primary analysis, however, does not support an increase in the incidence of dementia, therefore, another possible explanation is that due to a possible reduction in the ability to diagnose MCI after the protective measures were introduced people appeared to go straight from normal cognition rather than being diagnosed with MCI resulting in a reduction of progression from MCI to dementia. The CDR sum of boxes is more sensitive than the CDR global score but is not widely used to make clinical diagnoses. It is possible that the differences seen between our primary and secondary analyses are because of the greater sensitivity of the CDR sum of boxes. Another explanation is that the primary analysis failed to reject the null hypothesis due to, for example, the small sample size post‐exposure compared to the pre‐exposure group, loss of follow‐up, incorrect data collection or confounding factors. In a cohort study without random allocation of exposure to the risk factors a type II error may well be a possibility.^31^

In BDR, control interviews, even prior to the pandemic, were usually conducted over the phone. Individuals with MCI or dementia were usually seen face to face and the interviews contained additional questions. For individuals with more severe dementia (CDR global ≥3) interviews were with carers. Therefore, there was no change to control interviews due to the protective measures, but patient interviews were conducted remotely instead of face to face until at least mid 2021. Remote assessments may have been less sensitive to change or may have been affected by sensory impairment for example, hearing loss. This fact could have influenced our findings as subtle changes may have been missed resulting in more people not being recognised as having MCI at remote assessment and then being classified as having dementia at their next visit.

The COVID‐19 pandemic has led to changes in practice and limited the opportunity to perform face‐to‐face assessments due to emergency protective measures against the virus.^32^ Telephone consultations to screen for MCI and dementia were already widely used prior to the pandemic and there are many different tests available^32^ with widespread heterogeneity in research settings as result.^33^ Moreover, the TICS‐m test, for example, showed a lower sensitivity and specificity on detecting patients with MCI compared to patients with dementia, respectively 71% and 99% sensitivity, and 78% and 86% specificity.^33^, ^34^ Recently, a Cochrane review examining the diagnostic accuracy of remote cognitive assessments confirmed the heterogeneity in tests used and found limited supporting evidence to recommend one, single remote test. Despite this, the accuracy of telephone tests to diagnose dementia found in studies conducted after 2010 is promising with a sensitivity between 87% and 100%.^32^ These results are similar to in‐person tests such as MMSE and MoCA.^34^, ^35^

Although we could not examine this directly in our analysis due to lack of data, it is possible that one mechanism by which dementia incidence may have altered as a result of the pandemic is social isolation. Other possibilities include changes in other potentially modifiable risk factors such as physical activity, alcohol use, diet, and smoking. The association between social isolation and the risk of developing dementia has been studied extensively.^36^ Three systematic reviews and meta‐analyses looked at social isolation and concluded that people were more likely to develop dementia if social contact and social participation were neglected.^36^, ^37^, ^38^ A more recent study suggested that social isolation is associated with an increased risk of dementia even accounting for genetic risk.^39^ Similarly, there is evidence of an association between social isolation but not loneliness and an increased risk of developing dementia.^40^, ^41^ Loneliness or perceived isolation is the feeling of being socially and emotionally isolated, which can be present despite frequent social contact. Despite loneliness being frequently studied in relation to dementia, there remains controversy.^30^, ^37^, ^40^, ^41^, ^42^, ^43^, ^44^ Technology may be a promising tool in the prevention of social isolation or loneliness found during this pandemic,^45^ although this was not supported by a rapid review finding limited evidence of video calls to reduced loneliness in older adults and no studies reporting on video calls to help reduce social isolation.^46^ Co‐resident carers, on the other hand, have been shown to have a positive impact on people with dementia by increasing the quality of life (QoL) of both the patient and the carer, delaying admission to a care home and helping to maintain the relationship with the family.^47^

Strengths of our study include the longitudinal nature of the BDR data, the detailed data collected on potential confounders, the continuation of data collection during the protective measures against COVID‐19 and the availability of several different markers of cognitive impairment. We were also able to control for the effects of SARS‐CoV‐2 infection, although this data was collected less systematically than other variables. We examined whether individuals categorised using the CDR global score as having dementia had a clinical diagnosis, had reported memory problems and their MMSE/MOCA scores. Limitations include the large amount of missing data for the MMSE and MoCA, which are more widely used to define dementia than the CDR, relatively low numbers who developed dementia after lockdown, the relatively high education of this cohort compared to the UK older adult population due to the exclusion criteria used during the BDR cohort selection process, lack of data on social contacts (only available at baseline) and the potential issues with generalisability to other nations including relatively less advanced, low income countries and other healthcare settings.^48^ The CDR global score's main limitation in defining MCI is that using the CDR score of 0.5 for MCI can result in a heterogenous group ranging from healthy adults to mild dementia if not taking into account other factors or scoring systems, such as activities of daily living or MoCA.^49^ People may also have been isolating for a significant period prior to the formal lockdown and COVID‐19 was already in the UK, which would influence the number of patient progression to dementia related to the effects of the COVID‐19 pandemic, however, this factor is extremely difficult to verify. The cut‐off in March 2020, however, as starting lockdown date instead of the middle of March has been used in another study.^50^ Data was not available or insufficient on physical activity, co‐resident carer relationships or the number of social contacts making it difficult for us to comment on the mechanism by which lockdown may have exerted its effects. Surprisingly, the DETERMIND study group found evidence that the QoL of people who were recently diagnosed with dementia increased during the pandemic, which may have been due to them getting used to the diagnosis in a protected environment.^50^

Our data are interval‐censored because the exact date of the event within each interval is unknown. Methods for interval‐censored data with delayed entry are not yet available in standard software but our approximation of assuming the event to have occurred at the interval midpoint should be reasonable given the short duration of the intervals (median 425 days) relative to the age range of follow‐up (45–105 years). Age is a very important risk factor for dementia incidence, and it was necessarily associated with our exposure because the post‐lockdown period came after the pre‐lockdown period. Since the exact event date is unknown, there would be little advantage in adjusting for age more finely than the interval midpoint age used here, but our use of cubic splines for this allows a good fit to the observed association with age. The results, however, should be interpreted with caution given that a change in the way age was adjusted for in the secondary analysis related to MCI had a noticeable effect. It should also be noted that dementia develops over time. If the pandemic were promoting the development of dementia, an increase in incidence might not be immediately apparent but emerge long‐term with increasing duration of exposure.

## 5 CONCLUSION

In summary we found evidence to suggest that whilst the protective measures may not have led to an increase in dementia cases, we did find evidence to suggest that they may have led to more rapid deterioration in cognition for those who go on to develop dementia. Although the results should be interpreted with caution, this adds to the mounting evidence that the COVID‐19 pandemic is likely to have a long tail of increased mental health difficulties.^51^, ^52^ Future studies should include large numbers of individuals from a wider range of backgrounds to increase study power and generalisability and examine possible mechanisms by which the lockdown may have altered dementia development.

## AUTHOR CONTRIBUTIONS

Lindsey Isla Sinclair and Elizabeth Coulthard had the original idea. Lindsey Isla Sinclair, Daniel Hendrik Baron and Carslake David and performed the analysis and designed the analysis strategy with input from Elizabeth Coulthard. Daniel Hendrik Baron wrote the first draft of the paper which all authors contributed to. All authors have approved the final version of the manuscript and take responsibility for its scientific integrity.

## CONFLICT OF INTEREST STATEMENT

Lindsey Isla Sinclair is funded by a junior fellowship from the Alzheimer's Society, the Elizabeth‐Blackwell institute for Health Research University of Bristol and the Wellcome Trust Institutional Strategic Support Fund (204813/Z/16/Z). Carslake David works in a unit funded by the UK Medical Research Council (MC_UU_00011/1) and by the University of Bristol. Elizabeth Coulthard is funded by NIHR, Roestrees, Biogen, Above and Beyond, and BRACE charity and co‐investigator on MRC and EPSRC grants. Daniel Hendrik Baron has no declarations of interest to declare.

## Supporting information

Supporting Information S1

Figure S1

Figure S2

Table S1

Table S2

Table S3

Table S4

## Data Availability

The data that support the findings of this study are available from https://www.dementiasplatform.uk/ Restrictions apply to the availability of these data, which were used under licence for this study. DPUK is an MRC funded resource which is free for the dementia research community to use for approved research projects. More detailed information on DPUK and the cohorts included in this resource is available at https://www.dementiasplatform.uk/research‐hub/data‐portal.
