# Supplementary material for: The risk of developing dementia in the COVID‐19 pandemic; a cohort study
Source: Int J Geriatr Psychiatry. 2024 Jan 13;39(1):e6041. doi: 10.1002/gps.6041 (PMC10952166; doi:10.1002/gps.6041)
Supplement: Supplementary file 1 — Supporting Information S1 [file GPS-39-0-s005.docx]

**Descriptive statistics**

The chi-square test was used for categorical descriptive variables. Quantitative variables were first analysed for normal distribution. If the variable was normally distributed or could be transformed to obtain a normal distribution the one-way ANOVA-test was used. For variables where it was not possible to obtain a normal distribution the Kruskal-Wallis’ test was used.

**Adjusted risks factors**

We also adjusted for well-known risk factors for dementia such as hypertension and diabetes as well as potential risk factors for dementia, including marital status and accommodation, which differed between the groups. Unfortunately, only baseline data were available for social contacts making it impossible to directly assess the effects of social isolation on dementia risk.

**Detailed statistical description**

Peri-lockdown intervals were excluded from the analysis to investigate the effect of MCI on dementia incidence before and after lockdown, so the exposure became a simple binary variable distinguishing pre-lockdown and post-lockdown intervals. Separate incidence rate ratios for MCI for pre- and post-lockdown intervals were extracted from this model and the test of the interaction coefficient was interpreted as evidence of any difference between the two IRRs. This analysis was run once with a common effect of age before and after lockdown and once with independent effects of age.
