# Supplementary material for: The risk of developing dementia in the COVID‐19 pandemic; a cohort study
Source: Int J Geriatr Psychiatry. 2024 Jan 13;39(1):e6041. doi: 10.1002/gps.6041 (PMC10952166; doi:10.1002/gps.6041)

Supplementary figure 2: The effect of age in the crude secondary analysis using CDR sum of boxes to define outcome

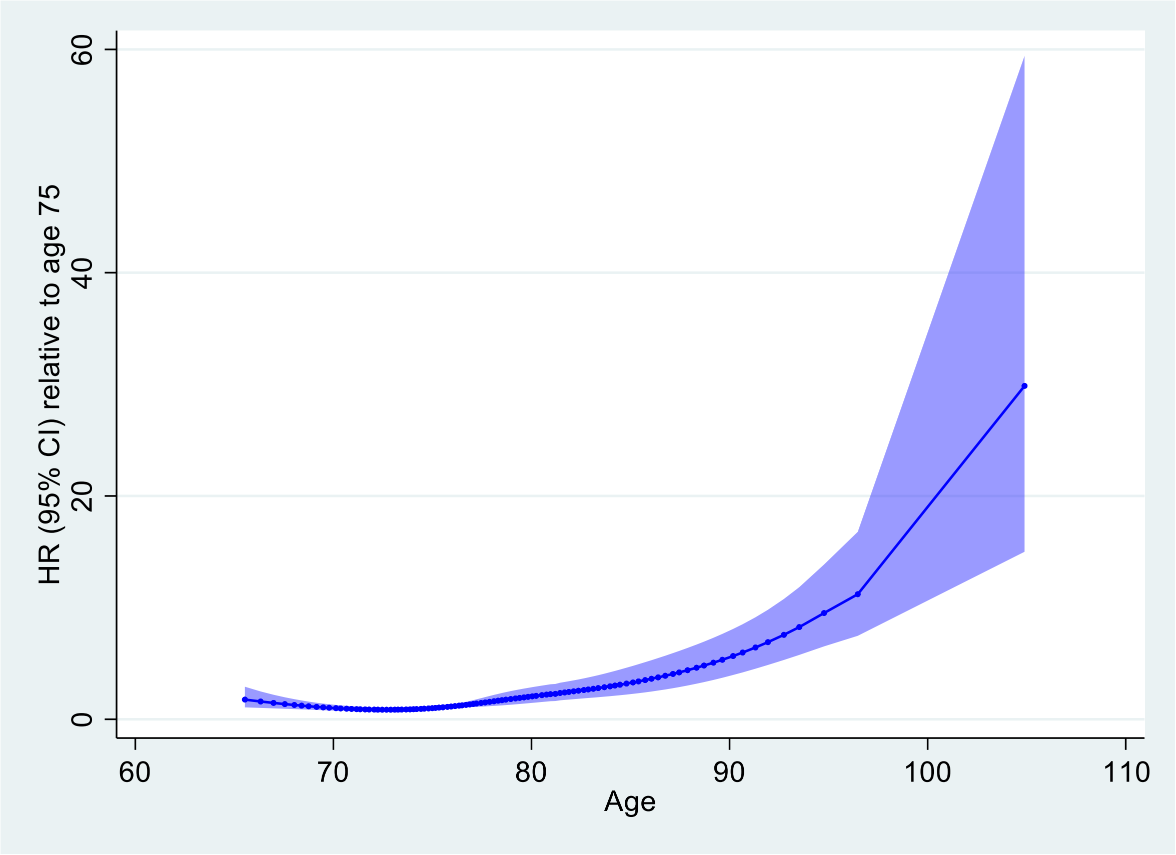

Supplement: Supplementary file 3 — Figure S2 [file GPS-39-0-s007.pdf]
