# Supplementary material for: The risk of developing dementia in the COVID‐19 pandemic; a cohort study
Source: Int J Geriatr Psychiatry. 2024 Jan 13;39(1):e6041. doi: 10.1002/gps.6041 (PMC10952166; doi:10.1002/gps.6041)
Supplement: Supplementary file 4 — Table S1 [file GPS-39-0-s004.pdf]

Supplementary Table 1: BDR participants entry and exclusion criteria

Entry criteria	Exclusion criteria
Brain donation after their demise	Brain trauma/ major stroke<br>Inadequate command of the English language to be able to complete assessments<br>Healthy controls aged <65 (except where partner of participant)<br>"Being geographically too remote from an assessment centre"

BDR is a collaboration between 6 major dementia research centres located at the Universities of King's College London, Oxford, Bristol, Newcastle, Manchester, and Cardiff. It is funded by Alzheimer's Research UK and the Alzheimer's Society and participants were recruited using advertisements in a wide range of media.
