# Supplementary material for: The risk of developing dementia in the COVID‐19 pandemic; a cohort study
Source: Int J Geriatr Psychiatry. 2024 Jan 13;39(1):e6041. doi: 10.1002/gps.6041 (PMC10952166; doi:10.1002/gps.6041)
Supplement: Supplementary file 5 — Table S2 [file GPS-39-0-s001.pdf]

Supplementary Table 2: The CDR global score (five-point scale) testing different cognitive and behavioural domains)

**CDR global score**	**Cognitive impairment**
0	None
0.5	Questionable
1	Mild
2	Moderate
3	Severe
