# Supplementary material for: The risk of developing dementia in the COVID‐19 pandemic; a cohort study
Source: Int J Geriatr Psychiatry. 2024 Jan 13;39(1):e6041. doi: 10.1002/gps.6041 (PMC10952166; doi:10.1002/gps.6041)
Supplement: Supplementary file 6 — Table S3 [file GPS-39-0-s003.pdf]

Supplementary Table 3: The independent effect of the risk of the covariates used in the analyses on the risk of conversion to dementia throughout the study follow-up.

	**Crude analysis IRR**	**Statistical evidence**	**Adjusted for age**	**Statistical evidence**
Sex *male vs female*	1.990 (1.511 to 2.621)	p<0.001	2.015 (1.528 to 2.658)	p<0.001
Sensory impairment *(severe versus normal)*	4.127 (1.711 to 9.951)	p=0.002	3.129 (1.289 to 7.596)	p=0.012
Covid infection	2.692 (1.380 to 5.251)	p=0.004	2.658 (1.356 to 5.210)	p=0.004
Stroke	3.393 (2.195 to 5.245)	p<0.001	2.846 (1.838 to 4.407)	p<0.001
Hypertension	1.140 (0.859 to 1.511)	p=0.364	1.008 (0.758 to 1.341)	p=0.956
Diabetes	1.400 (0.920 to 2.130)	p=0.116	1.412 (0.926 to 2.152)	p=0.109
Marital status				
Divorced vs married	0.342 (0.159 to 0.737)	p=0.006	0.306 (0.142 to 0.662)	p=0.003
Single vs married	0.224 (0.082 to 0.609)	p=0.003	0.161 (0.059 to 0.439)	p<0.001
