# Supplementary material for: The risk of developing dementia in the COVID‐19 pandemic; a cohort study
Source: Int J Geriatr Psychiatry. 2024 Jan 13;39(1):e6041. doi: 10.1002/gps.6041 (PMC10952166; doi:10.1002/gps.6041)
Supplement: Supplementary file 7 — Table S4 [file GPS-39-0-s002.pdf]

Supplementary Table 4: Missing data at baseline

Variable	No. Missing	Number with data	Unique Values
Age at baseline	0		
Gender	0		
Education	0		
Smoking	0		
CDR sum of boxes	0		
Smoking	0		
Drinking	0		
Diabetes	0		
Family History	0		
Cancer	0		
SARS-CoV-2 infection	0		
SARS-CoV-2 vaccine	0		
Head injury	0		
Hypertension	0		
Heart attack	0		
BALD at baseline	1372	901	24
HICS at baseline	1651	622	10
MMSE at baseline	194	2019	18
MOCA at baseline	1042	1231	19
TICS at baseline	2247	26	13
Geriatric Depression Scale at baseline	168	2105	15
Marital Status at baseline	38	2235	5
Accommodation at baseline	287	1986	7
Memory Problem at baseline	1907	366	3

Sensory impairment at baseline	619	1654	5
Stroke	6	2267	2
